# Clinico-histopathologic and single-nuclei RNA-sequencing insights into cardiac injury and microthrombi in critical COVID-19

**DOI:** 10.1172/jci.insight.154633

**Published:** 2022-01-25

**Authors:** Michael I. Brener, Michelle L. Hulke, Nobuaki Fukuma, Stephanie Golob, Robert S. Zilinyi, Zhipeng Zhou, Christos Tzimas, Ilaria Russo, Claire McGroder, Ryan D. Pfeiffer, Alexander Chong, Geping Zhang, Daniel Burkhoff, Martin B. Leon, Mathew S. Maurer, Jeffrey W. Moses, Anne-Catrin Uhlemann, Hanina Hibshoosh, Nir Uriel, Matthias J. Szabolcs, Björn Redfors, Charles C. Marboe, Matthew R. Baldwin, Nathan R. Tucker, Emily J. Tsai

**Affiliations:** 1 Division of Cardiology, Columbia University Irving Medical Center (CUIMC), New York, New York, USA.; 2 Masonic Medical Research Institute, Utica, New York, USA.; 3 Department of Medicine, CUIMC, New York, New York, USA.; 4 Cardiovascular Research Foundation, New York, New York, USA.; 5 Division of Pulmonary, Allergy & Critical Care Medicine,; 6 Division of Infectious Diseases, and; 7 Department of Pathology and Cell Biology, CUIMC, New York, New York, USA.; 8 St. Francis Hospital Heart Center, Roslyn, New York, USA.; 9 Cardiovascular Disease Initiative, Broad Institute of MIT and Harvard, Cambridge, Massachusetts, USA.

**Keywords:** COVID-19, Cardiology, Bioinformatics, Cardiovascular disease, Molecular pathology

## Abstract

Acute cardiac injury is prevalent in critical COVID-19 and associated with increased mortality. Its etiology remains debated, as initially presumed causes — myocarditis and cardiac necrosis — have proved uncommon. To elucidate the pathophysiology of COVID-19–associated cardiac injury, we conducted a prospective study of the first 69 consecutive COVID-19 decedents at CUIMC in New York City. Of 6 acute cardiac histopathologic features, presence of microthrombi was the most commonly detected among our cohort. We tested associations of cardiac microthrombi with biomarkers of inflammation, cardiac injury, and fibrinolysis and with in-hospital antiplatelet therapy, therapeutic anticoagulation, and corticosteroid treatment, while adjusting for multiple clinical factors, including COVID-19 therapies. Higher peak erythrocyte sedimentation rate and C-reactive protein were independently associated with increased odds of microthrombi, supporting an immunothrombotic etiology. Using single-nuclei RNA-sequencing analysis on 3 patients with and 4 patients without cardiac microthrombi, we discovered an enrichment of prothrombotic/antifibrinolytic, extracellular matrix remodeling, and immune-potentiating signaling among cardiac fibroblasts in microthrombi-positive, relative to microthrombi-negative, COVID-19 hearts. Non–COVID-19, nonfailing hearts were used as reference controls. Our study identifies a specific transcriptomic signature in cardiac fibroblasts as a salient feature of microthrombi-positive COVID-19 hearts. Our findings warrant further mechanistic study as cardiac fibroblasts may represent a potential therapeutic target for COVID-19–associated cardiac microthrombi.

## Graphical abstract

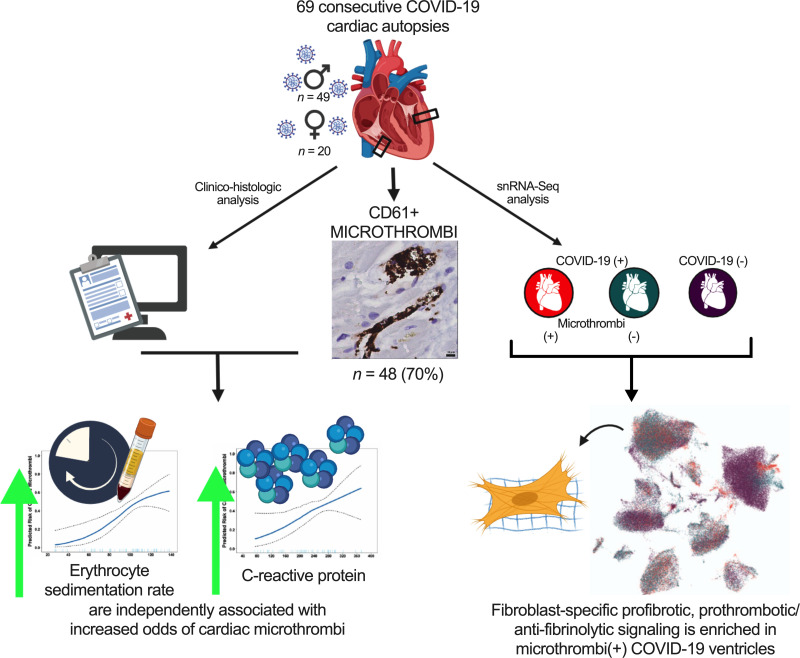

## Introduction

In severe COVID-19, cardiac manifestations like arrhythmias, myocardial infarction, acute heart failure, and cardiogenic shock are prevalent and associated with increased mortality (1–8). Both direct and indirect mechanisms of COVID-19–associated cardiac injury have been postulated (2), yet details remain elusive. Early theories that COVID-19 cardiac injury reflected acute viral myocarditis have since been disproved by the rarity of cases that meet histologic criteria for myocarditis (9). Additionally, diverse cardiac histopathologic findings, including endothelial cell damage and inflammatory cell infiltrates without associated cardiac necrosis (10–12), have been described on autopsy of COVID-19 decedents but at variable prevalence. Subsequently, the detection of microvascular thrombi in the hearts (13, 14), lungs (15–19), liver (20), brain (21), and skin (22) of patients with COVID-19 raised the possibility that, in severe cases, COVID-19 may be more akin to a systemic illness. Such reports of multiorgan microthrombi, along with a high incidence of venous thromboembolism in severe and critical COVID-19, have led investigators to hypothesize that severe SARS-CoV-2 infection promotes a hypercoagulable state, referred to as COVID-19–associated coagulopathy (23–27). However, clinical trials revealed that neither empiric intermediate-dose prophylactic anticoagulation nor therapeutic anticoagulation for critical COVID-19 reduce thrombosis, the need for cardiovascular or respiratory organ support, or mortality (28–30). Bench studies and clinical observations suggest that thrombosis in COVID-19 may be primarily immune mediated, and therefore, targeting immunothrombosis may be essential to prevent or treat thrombotic complications (31, 32). Clinical, patient-oriented studies have yet to test this hypothesis.

Whether cardiac microthrombi underlie acute cardiac injury in severe COVID-19 is unclear at this time. Early COVID-19 cardiac autopsy series focused on detecting acute viral myocarditis and the SARS-CoV-2 virion. As such, the prevalence of cardiac microthrombi in fatal COVID-19 is not well established. Moreover, most COVID-19 cardiac autopsy studies were limited to sample sizes fewer than 50 persons (10, 13, 14, 17, 18, 33–35). Many had long postmortem intervals (PMIs, i.e., the time between death and autopsy) (13, 33–36). Long PMIs have been shown to compromise tissue RNA integrity and yield (37), particularly that of the heart (38), thereby hindering molecular analyses of postmortem hearts in prior COVID-19 studies.

To investigate the molecular pathophysiology of cardiac injury in fatal COVID-19, we instituted specific measures to reduce PMI and optimize autopsy specimen quality for immunohistology and single-nuclei RNA sequencing (snRNA-Seq). We examined both left and right ventricular cardiac tissue samples of COVID-19 decedents and determined the prevalence of acute cardiac histopathologic features attributable to SARS-CoV-2 infection, including cardiac microthrombi, within our cohort. We analyzed patient clinical data, ventricular viral load, and histopathologic features of each heart to identify clinico-histopathologic associations that could provide pathophysiologic insights into COVID-19–associated cardiac injury. Finding microthrombi to be the most common acute cardiac histopathology in our study cohort, we subsequently conducted snRNA-Seq analysis of a subset of COVID-19 cardiac tissue with and without microthrombi to determine the compositional and cell type–specific transcriptomic changes that may underlie the development of COVID-19–associated cardiac microthrombi.

## Results

### Patient characteristics.

Our study cohort of 69 laboratory-confirmed COVID-19 decedents had a mean age of 72 ± 10 (range 38–97) years and 20 (29%) were women. Decedents were predominantly Hispanic (59%) or Black (16%). Hypertension, diabetes, and obesity were the most common comorbidities. The median time from hospitalization to death was 16 [IQR 4–36] days. Forty-three patients (62%) received at least 1 form of critical care intervention, with 41 (59%) patients supported on mechanical ventilation for 13 [IQR 2–34] days, 37 (54%) patients receiving vasoactive medication, and 15 (22%) patients receiving renal replacement therapy. Of the 28 patients not mechanically ventilated, all had hypoxemic respiratory failure but either requested do not resuscitate/do not intubate orders (33%) or died during attempted cardiopulmonary resuscitation (7%). Nearly 60% of patients received corticosteroids; far fewer received tocilizumab (16%) or remdesivir (3%). Peak high-sensitivity cardiac troponin T (hs-cTnT) exceeded 99th percentile reference limits in 93% of decedents; median peak hs-cTnT was 99 [IQR 47–227] ng/L. D-dimer, erythrocyte sedimentation rate (ESR), and C-reactive protein (CRP) were also markedly elevated (Table 1). All autopsies were performed with a median PMI of 20 [IQR 5.5–42.5] hours; 20 autopsies had PMI less than 8 hours.

### Cardiac histopathology.

**Figure 1:**
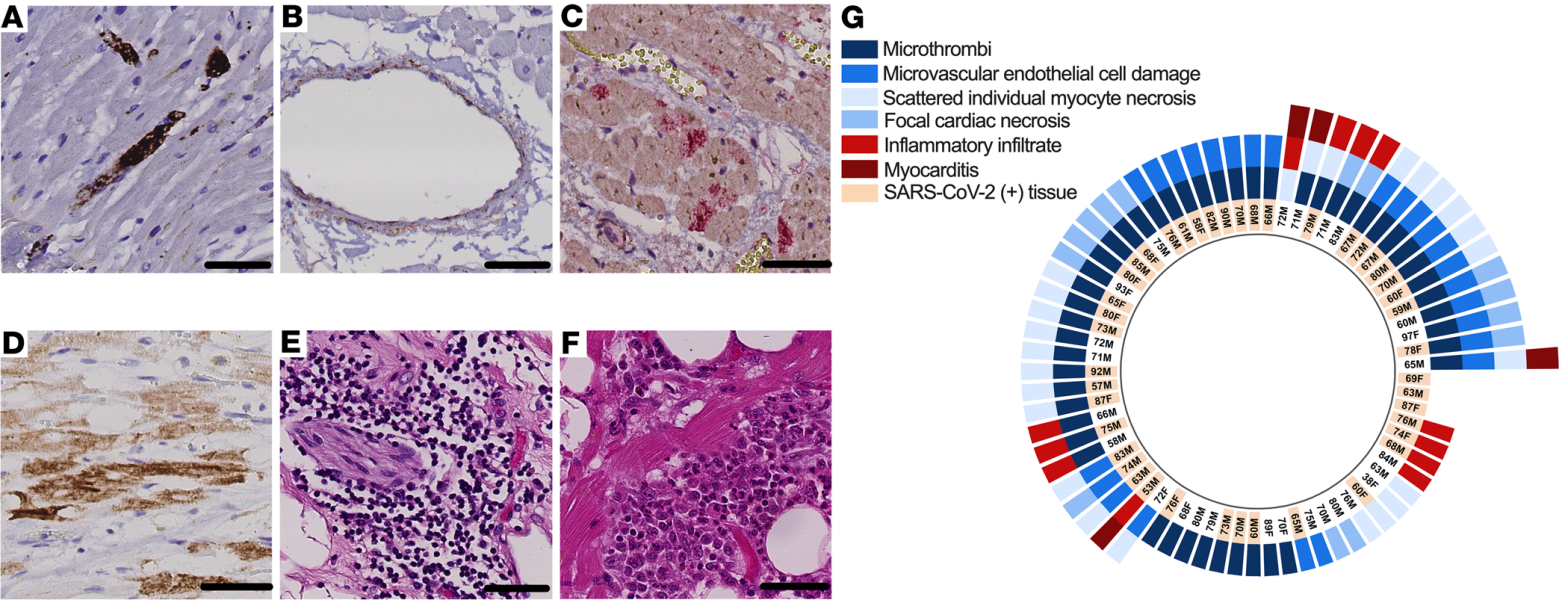
Acute cardiac histopathologic features in fatal COVID-19. (**A**) Microthrombi, identified by intraluminal CD61 immunostaining (DAB chromogen, brown) of intramyocardial small vessels. (**B**) Small vascular endothelial cell damage, identified by C4d immunostaining (DAB chromogen, brown) of vascular endothelial cells. (**C**) Focal cardiac necrosis, identified by a confluence of C4d^+^ cardiomyocytes (DAB chromogen, brown) covering an area of at least 0.05 mm^2^ but less than or equal to 1 cm^2^. (**D**) Inflammatory infiltrates on H&E. (**E**) Scattered, isolated cardiomyocyte necrosis, identified by C4d^+^ staining (Alkaline Phosphatase chromogen, red) of cardiomyocytes. (**F**) Focal myocarditis on H&E. Scale bars: 50 μm. (**G**) Radial plot of acute cardiac histopathologic features detected in either ventricle of each COVID-19 decedent’s heart. Detection by quantitative reverse transcription polymerase chain reaction (RT-qPCR) of SARS-CoV-2 in cardiac tissue, where decedents’ age and sex are also shown.

Upon microscopic examination of both left and right ventricular tissue samples of the 69 hearts, we detected at least 1 of 6 acute cardiac histopathologic features in nearly all decedents (*n* = 67, 97%): microthrombi (*n* = 48, 70%), microvascular endothelial cell damage (*n* = 25, 36%), scattered individual cardiomyocyte necrosis (*n* = 25, 36%), focal cardiac necrosis without any adjacent inflammatory infiltrate (*n* = 14, 20%), focal inflammatory infiltrates without associated cardiomyocyte injury (*n* = 12, 17%), and focal myocarditis (*n* = 4, 6%) (Figure 1). Microthrombi were detected in serial sections by immunostaining for platelets (CD61). Microvascular endothelial cell damage was identified in serial sections by complement protein C4d immunostaining and by endothelial cell marker CD31 immunostaining, both with hematoxylin counterstain. Necrotic cardiomyocytes were likewise identified by C4d immunostaining, and their characteristic branching, striated morphology was appreciated on hematoxylin counterstain. Focal cardiac necrosis was defined as a confluence of adjacent C4d^+^ cardiomyocytes occupying an area greater than 0.05 mm^2^ but not exceeding 1 cm^2^. Inflammatory infiltrates were detected by H&E staining. Most hearts (*n* = 49, 71%) exhibited 2 or more of these acute histopathologic features, including microthrombi in 39 (80%) of such cases (Figure 1G). Macroscopic and other histopathologic autopsy findings are summarized in Supplemental Table 1; supplemental material available online with this article; https://doi.org/10.1172/jci.insight.154633DS1

Presence of cardiac microthrombi was the most common of the 6 acute cardiac histopathologic findings detected in our study cohort. Hence, we deemed cardiac microthrombi the major histologic phenotype of acute cardiac injury in critical COVID-19. We then examined the association between the other acute cardiac histopathologic findings and cardiac microthrombi to explore potential pathogenic mechanisms. In analyses adjusting for age and sex, microvascular endothelial damage was associated with cardiac microthrombi in the corresponding ventricle (OR 3.58, 95% CI 1.46–8.80). Cardiomyocyte necrosis, focal necrosis, inflammatory infiltrate, and myocarditis were not independently associated with cardiac microthrombi (Supplemental Table 2).

### Ventricular viral load.

We detected SARS-CoV-2 by RT-qPCR in 43 (62%) hearts. In analyses adjusting for age and sex, ventricular viral load was nonsignificantly associated with higher odds of microthrombi in the corresponding ventricle (Supplemental Table 3). In contrast, detectable SARS-CoV-2 in ventricular tissue was independently associated with microvascular endothelial cell damage in the corresponding ventricle (OR 2.36, 95% CI 1.04–5.35).

### Biomarkers and clinical risk factors.

**Figure 2:**
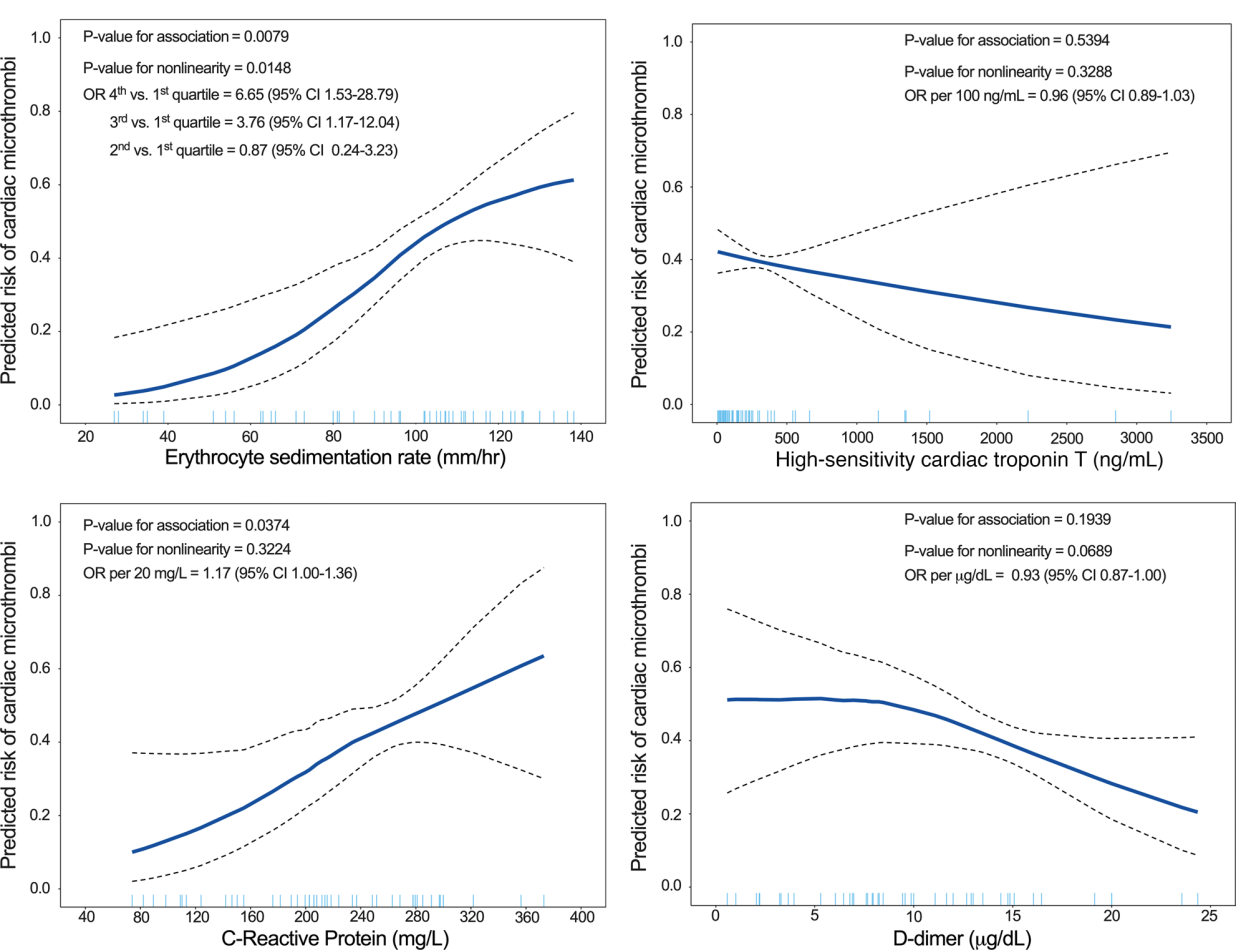
Adjusted GAMs of the association between serum biomarker peak values and cardiac microthrombi. We calculated a covariate balancing propensity score (CBPS) for each independent variable by regressing it on possible confounders; the resulting propensity score was used in the GAM as a single covariable. The covariates used to calculate CBPS were age, sex, race and ethnicity, BMI, duration of COVID-19 illness, outpatient ACE inhibitor/angiotensin receptor blocker (ARB) use, outpatient antiplatelet therapy, and inpatient administration of corticosteroids, remdesivir, interleukin-6 (IL-6) receptor antagonists, and therapeutic anticoagulation.

Given the hypothesized immunothrombotic etiology of cardiac microthrombi, we tested the associations of biomarkers of cardiac injury, fibrinolysis, and inflammation with the presence of cardiac microthrombi in COVID-19 hearts. In generalized additive logistic models (GAMs) adjusted for demographic, clinical, outpatient medication, COVID-19 treatment, and inpatient anticoagulation factors, peak hs-cTnT and D-dimer were not associated with the predicted risk of cardiac microthrombi. However, peak CRP was linearly associated with the predicted risk of cardiac microthrombi. ESR varied nonlinearly with predicted risk of cardiac microthrombi, with plateaus of risk at levels less than 60 and more than 100 mm/h (Figure 2). In fully adjusted logistic regression models, every 20 mg/L change in CRP was associated with 1.17 (95% CI 1.00–1.36) higher odds of cardiac microthrombi. Comparing patients in the third and fourth quartiles of ESR (107–126 and 130–169 mm/h) with those in the first quartile (27–80 mm/h), there were 3.76 (95% CI 1.17–12.04) and 6.65 (95% CI 1.53–28.79) higher odds of cardiac microthrombi, respectively (Supplemental Table 4).

In models adjusted for demographic, clinical, and treatment factors, use of outpatient antiplatelet therapy and use of in-hospital therapeutic anticoagulation were nonsignificantly associated with lower odds of cardiac microthrombi, whereas in-hospital corticosteroid therapy was nonsignificantly associated with higher odds of cardiac microthrombi (Supplemental Figure 1). Although more common among the COVID-19 decedents with cardiac microthrombi, outpatient use of ACE inhibitor/ARB was not independently associated with cardiac microthrombi after multivariable adjustment (Table 1 and Supplemental Figure 1).

Vasoactive therapy and mechanical ventilation were more commonly used in COVID-19 decedents without cardiac microthrombi than in those with cardiac microthrombi, possibly reflecting differences in clinical phenotypes, patient-directed goals of care, or both. Moreover, without biological rationale for antithrombotic action, neither vasoactive therapy nor mechanical ventilation was included in multivariable-adjusted GAMs as a covariate.

### snRNA-Seq.

To gain insight into the molecular pathophysiology of COVID-19–associated cardiac microthrombi, we performed snRNA-Seq on mid-right ventricular free wall tissue from 8 COVID-19 decedents with very low PMI (3.5 [IQR 2.8–5.0] hours): 4 with and 4 without microthrombi (Supplemental Table 5). No SARS-CoV-2 transcripts were detected in any sample — an expected reflection of the known non-nuclear, cytosolic residence of SARS-CoV-2. We excluded 1 of the 4 microthrombi-positive samples from downstream analysis because of its low cellular complexity and high mitochondrial read count (Supplemental Table 6). We compared data from the microthrombi-positive (13,068 nuclei) and microthrombi-negative (30,425 nuclei) COVID-19 samples with each other and with data from 6 nonfailing hearts of the Human Cell Atlas (HCA) (39) as the non–COVID-19 reference cohort (32,279 nuclei). Generated as part of a global effort to provide a nonfailing reference map for analyses such as ours, the HCA heart reference has the potential to yield technically driven effects. To minimize such potential, we reanalyzed the HCA reference from raw reads in parallel with our COVID-19 samples. Perfectly confounded, the HCA reference cohort could not be adjusted for any differences in sample collection, sample processing, or library generation. Thus, results from COVID-19 versus HCA reference comparison should be interpreted with this in mind. In contrast, microthrombi-positive and microthrombi-negative COVID-19 samples were collected and processed in parallel, and therefore results comparing them can be interpreted with greater confidence.

**Figure 3:**
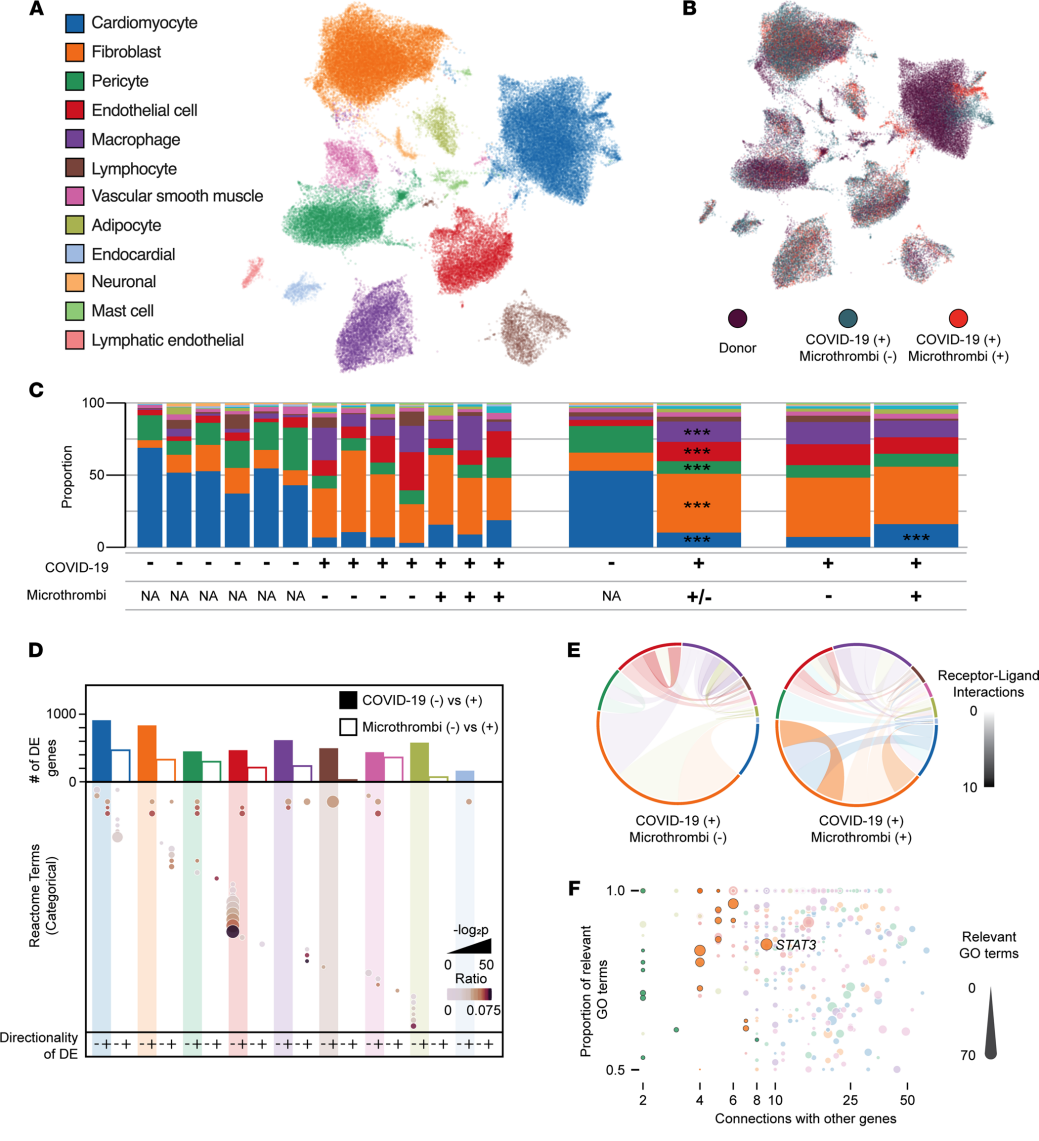
snRNA-Seq of right ventricular tissue from COVID-19 autopsies. (**A**) Uniform manifold approximation and projection (UMAP) of 75,772 individual nuclei colored by cell type as determined from marker gene analysis. Colors observed within this legend are used throughout the figure. (**B**) UMAP colored by sample type of origin. (**C**) Compositional analysis for each cell type by individual sample, COVID-19 status, and microthrombi detection. *** indicates a credible increase or decrease in cell proportion when compared with the referent data set. (**D**) Bar plot displays the total number of DE genes at 0.25 LFC for COVID-19 versus non–COVID-19 control (filled bar) or microthrombi-positive versus microthrombi-negative in the COVID-19 (+) samples (unfilled bar). Dot plot indicates the Reactome terms associated with DE genes in a given comparison and direction. Size and shade of the dot represent the adjusted *P* value and ratio of Reactome genes present for a given term. (**E**) CellPhoneDB analysis of cell-cell communication through analysis of receptor-ligand pair expression. Size of each outer section corresponds to the proportion of that cell type in the tissue. Opacity of each connection indicates the number of connections between cell types (paracrine and autocrine). Color of the connection indicates the cell type producing the ligand. (**F**) Dot plot of GeneWalk analysis identifying regulator genes for the ontology observed within the microthrombi-positive versus microthrombi-negative comparison within the COVID-19 (+) samples. Color corresponds to the cell type, and size of the dot represents the number of significant Gene Ontology terms affiliated with that gene. Dots with high opacity represent those from the 0.5 LFC threshold for DE genes, while those with reduced opacity result from the more liberal 0.25 LFC threshold.

Principal components analysis of sample-level transcript counts clearly separated microthrombi-positive from microthrombi-negative COVID-19 samples and COVID-19 from non–COVID-19 reference samples (Supplemental Figure 2). After stringent quality control measures (Supplemental Figure 3), we analyzed the cellular diversity and transcriptional signatures of the aggregated snRNA-Seq data, across all patient subsets (Figure 3). We identified 12 distinct cell types (Figure 3A), consistent with recent analyses of nonfailing human myocardium (39, 40). Sensitive and specific transcriptional markers for each cell type are listed in Supplemental Table 7.

To examine whether cell composition differs in COVID-19 versus non–COVID-19 reference hearts or in microthrombi-positive versus microthrombi-negative COVID-19 hearts, we quantified and compared relative proportions of each cell type in our snRNA-Seq data, for each of the comparison groups (Figure 3, B and C). Any differences in cell composition could reflect either direct or indirect effects of SARS-CoV-2 infection, whereby the magnitude of difference may represent the relative role of or effect upon specific cell types in COVID-19–associated cardiac injury and cardiac microthrombi. We identified credible differences in cell type proportions in COVID-19 versus reference samples — decreases in cardiomyocytes (–2.33 log_2_ fold change, LFC) and pericytes (–0.90 LFC) and increases in fibroblasts (1.67 LFC), endothelial cells (1.46 LFC), and macrophages (2.26 LFC) (Figure 3C and Supplemental Table 8). Microthrombi-negative COVID-19 samples exhibited greater loss of cardiomyocytes than did the microthrombi-positive COVID-19 subset (Supplemental Table 9).

We then identified the differentially expressed (DE) genes in the 9 most numerous cell types across subset comparisons of (a) COVID-19 versus non–COVID-19 reference control and (b) microthrombi-positive versus microthrombi-negative COVID-19 (Supplemental Figure 4 and Supplemental Table 10). We examined gene set enrichment in all DE genes for each subset comparison (Figure 3D and Supplemental Table 11). In COVID-19 versus reference control, multiple cell types shared several altered regulatory patterns. Upregulation of chromatin-modifying enzymes suggests epigenetic changes in all but endothelial cells, and altered Rho GTPases imply effects on cytoskeletal dynamics and cell motility in non-immune cells. We also uncovered cell type–specific features in COVID-19 samples compared with reference controls. In vascular endothelial cells, multiple pathways associated with IL-6 and interferon signaling were downregulated. By examining the DE genes that made up these pathways, we found significant and marked downregulation of HLA class I proteins (HLA-A, -B, and -C) and JAK/STAT family members — a response that has been observed as a signature of viral immune evasion (41). In cardiomyocytes and vascular smooth muscle, pathways involved in striated and smooth muscle contraction were respectively downregulated, driven by genes encoding structural components of contractile apparatuses (i.e., actinomyosin family members, troponins, and tropomyosins), signifying contractile dysfunction.

Cell type–specific signaling signatures were also noted in microthrombi-positive compared with microthrombi-negative COVID-19 specimens. In fibroblasts, extracellular matrix (ECM) pathways were markedly upregulated, as were multiple genes that encode different types of collagen and genes that encode proteins with dual antifibrinolytic/prothrombotic and innate immune response–related functions (e.g., *SERPINE1*, *THBS2*). In macrophages, cytokine signaling was upregulated. In cardiomyocytes, striated muscle contraction pathways were further downregulated, signifying more severe contractile dysfunction in microthrombi-positive COVID-19 hearts.

To assess cell-cell communication, we then examined the expression of receptor-ligand pairs by each cell type population (Figure 3E and Supplemental Table 12). The number of cell type–specific signaling interactions involving fibroblasts was notably greater in microthrombi-positive compared with microthrombi-negative COVID-19 samples. With some autocrine and others paracrine in nature, the additional fibroblast signaling interactions in microthrombi-positive samples involved expression of multiple collagen types, the α_11_β_1_ integrin complex, and the PDGF D and PDGF receptor complex.

We then used a network representation learning framework (42) to identify regulator genes — DE genes with the most connections to all other DE genes and to their gene ontologies. We searched for regulator genes that account for both the differences between COVID-19 and reference control (Supplemental Figure 5 and Supplemental Table 13) and those between microthrombi-positive and microthrombi-negative COVID-19 samples (Figure 3F). In sum, we identified 254 unique regulator genes, distributed across all cell types, that accounted for the differences between microthrombi-positive and microthrombi-negative COVID-19 samples (Supplemental Table 13). Nearly a third of these regulator genes were shared by multiple cell types, indicating that a common cellular response is specific to COVID-19–associated cardiac microthrombi at the tissue level. To identify regulator genes with larger effect sizes, we examined genes with greater differential expression (≥0.5 LFC) between the microthrombi-positive and microthrombi-negative COVID-19 subsets. We uncovered 17 such large-effect regulator genes in fibroblasts and 9 in pericytes but none in any other cell types (Figure 3F and Supplemental Table 13). Notably, *FGFR1* was one of the fibroblast-specific large-effect regulator genes, supporting the association of fibroblast proliferation and activation with the presence of microthrombi. Ontology terms underlying the connectivity of large-effect regulator genes revealed signaling associations including ECM deposition (collagen family members) and fibroblast activation (*STAT3*, *THBS1*) in fibroblasts (43, 44), further emphasizing that dysregulated fibroblast activity is a salient feature of COVID-19–associated cardiac microthrombi (Supplemental Figure 6).

We intersected large-effect regulator genes with druggable genome annotations (45) to prioritize targets for potential follow-up therapeutic investigation (Supplemental Table 13). For COVID-19 versus reference control comparisons, 23 large-effect regulator genes were categorized in the druggable genome as tier 1 (i.e., targeted by approved small molecules or late-stage drug candidates). Microthrombi-positive versus microthrombi-negative COVID-19 comparisons yielded 4 tier 1 targets: *PIP5K1A*, *STAT3*, and *VEGFA* in fibroblasts and *CSNK1A1* in pericytes.

**Figure 4:**
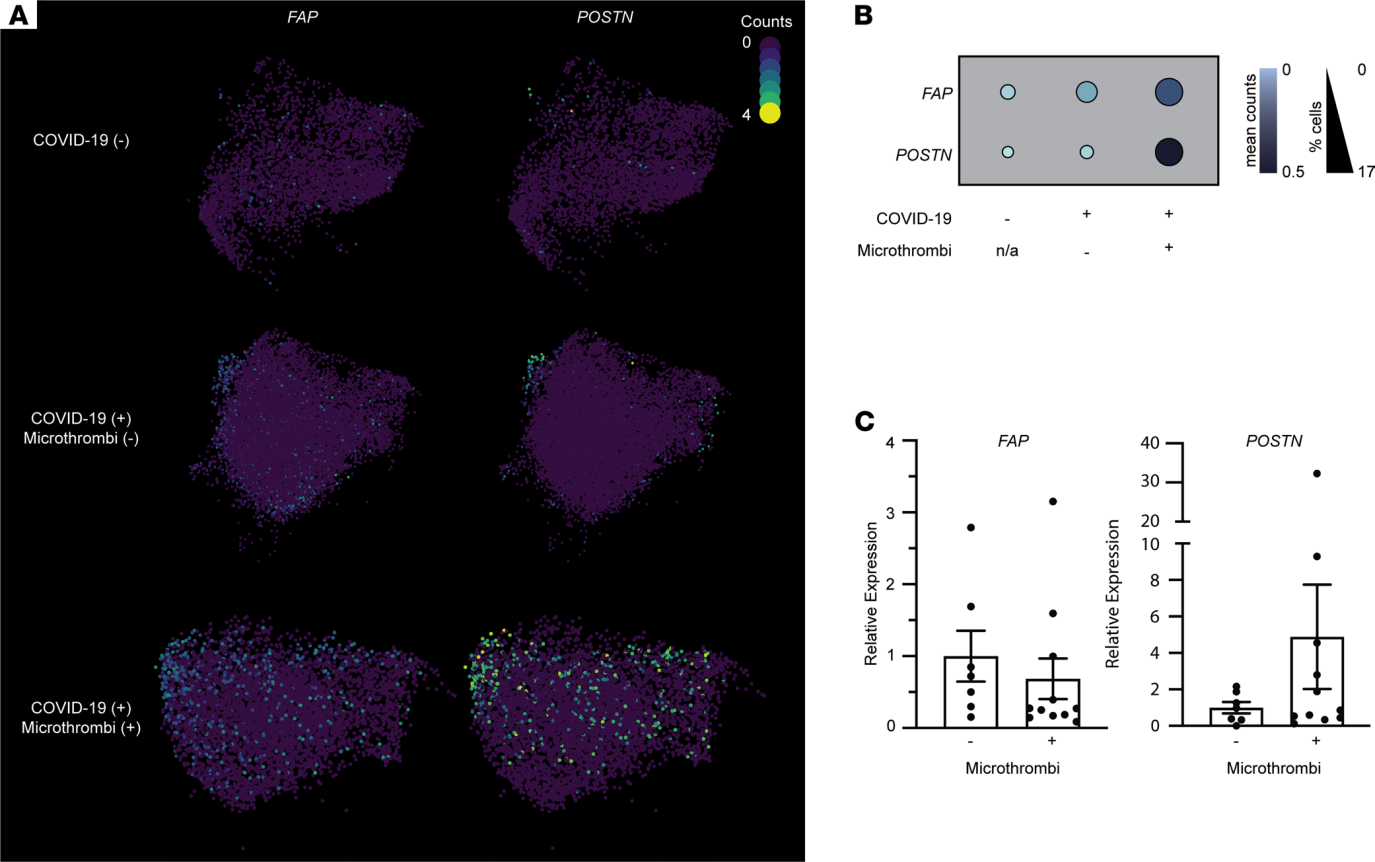
Analysis of fibroblasts’ activation in COVID-19 samples with versus without microthrombi. (**A**) UMAPs of fibroblast cell populations. Colors correspond to the read counts for *FAP* and *POSTN* in each cell. (**B**) Dot plot displaying the expression of activated fibroblast markers *FAP* and *POSTN* in fibroblast clusters. Size and shade of the dot correspond to the percentage of cells with non-zero counts and the mean counts across all cells, respectively. (**C**) Scatter plot of relative expression of *FAP* and *POSTN* (corrected to *GAPDH*), as measured by probe-based RT-qPCR, in ventricular tissue samples from COVID-19 autopsies with PMI less than 8 hours (same PMI criteria as used for snRNA-Seq). Bars represent mean ± SEM. *n* = 7 for microthrombi-negative; *n* = 11 for microthrombi-positive COVID-19 groups. All RT-qPCR samples were run in duplicate. *P* = 0.96 for *FAP*, *P* = 0.20 for *POSTN* on Welch’s *t* test.

Given the convergence of profibrotic fibroblast signatures, we wished to specifically examine the activation state of fibroblasts in microthrombi-positive COVID-19 samples. We examined the expression of canonical markers of fibroblast activation, fibroblast activating protein (*FAP*) (46) and periostin (*POSTN*) (47), in our snRNA-Seq data. The percentage of fibroblasts expressing these markers and the mean expression of these markers were higher in microthrombi-positive versus microthrombi-negative COVID-19 samples, but they were not expressed broadly enough to be included in our wider DE model (Figure 4, A and B). To examine this further, we measured the ventricular expression of *FAP* and *POSTN* in COVID-19 samples with PMI less than 8 hours (PMI criteria used for snRNA-Seq analysis). In the microthrombi-positive COVID-19 samples, myocardial expression of *POSTN* was nonsignificantly increased relative to that in microthrombi-negative samples (Figure 4C). *FAP* expression was comparable between the 2 groups. Expanded analysis of all COVID-19 samples with PMI less than 24 hours demonstrated similar results (Supplemental Figure 7); microthrombi-positive samples expressed nonsignificantly higher levels of *POSTN* and similar levels of *FAP* as microthrombi-negative samples.

## Discussion

By leveraging our single-center prospective study design and low-PMI autopsy protocol, we identified microthrombi — not cardiac necrosis, microvascular endothelial damage, or inflammatory infiltrates — as the predominant acute cardiac histopathology in fatal COVID-19. We substantiated its presumed immunothrombotic nature through clinico-histopathologic analyses, and, most significantly, identified a cell type–specific transcriptomic signature that suggests cardiac fibroblasts have a significant pathophysiologic role in cardiac injury due to COVID-19–associated cardiac microthrombi. While controlling for multiple clinical covariates, including in-hospital therapies, we found that biomarkers of systemic inflammation, but not those of fibrinolysis or cardiac injury, were independently associated with cardiac microthrombi. Furthermore, antiplatelet and anticoagulation therapy had a nonsignificant association with lower odds of cardiac microthrombi, suggesting that anticoagulation may be necessary but insufficient in preventing and treating cardiac microthrombi in critical COVID-19. We also found an association, independent of age and sex, between microvascular endothelial cell damage and microthrombi in the COVID-19 hearts. These clinico-histopathologic associations are consistent with the conventional model of immunothrombosis as a convergence of endothelial cell dysfunction, immune cell activation, and platelet activation (48). Most notably and unexpectedly, our snRNA-Seq analysis suggests that dysregulated cardiac fibroblasts expressing high levels of prothrombotic/antifibrinolytic, ECM-remodeling, and immune-potentiating genes are a salient feature of microthrombi-positive hearts in critical COVID-19.

Our study is distinct from prior single-cell/snRNA-Seq analyses of COVID-19 tissue specimens (49, 50) in 3 major aspects. First, by defining our COVID-19 subsets according to the presence of cardiac microthrombi, we optimized the translational significance of our snRNA-Seq analysis. The comparison of COVID-19 hearts with and without microthrombi provided an internal control for differences associated more generally with COVID-19. Any cell type–specific cellular and transcriptomic differences would relate more specifically to COVID-19–associated cardiac microthrombi, rather than COVID-19 itself. This distinction is particularly significant given that therapeutic anticoagulation does not reduce thrombotic events or improve the survival of critically ill COVID-19 patients (30). Analogous to the clinical trial findings on anticoagulation, our clinico-histopathologic analysis found that in-hospital administration of therapeutic anticoagulation was not independently associated with the presence (or absence) of cardiac microthrombi in our cohort. Therefore, our snRNA-Seq analysis could potentially suggest novel approaches for preventing or treating cardiac microthrombi in critical COVID-19. Second, by using immunohistology to systematically examine all ventricular samples for multiple prespecified histopathologic features, we could select snRNA-Seq samples to avoid potential confounding effects of coexisting histopathology. This also facilitated an unbiased approach to identifying the most prevalent cardiac histopathology in our cohort. We did not assume that acute cardiac injury was synonymous with cardiac necrosis or any other histopathology. We examined all samples for all histopathologic features of interest, thereby avoiding any selection bias that a hierarchical approach would introduce. Third, likely unique to our study, the granularity of our clinical data provided an invaluable framework for interpreting the snRNA-Seq data.

Additional strengths of our study include our cohort demographics and low PMI. Our study cohort consisted predominantly of Black and Hispanic decedents, reflecting their disproportionate share of COVID-19 deaths in the United States relative to their overall population (51). Yielding 20 COVID-19 autopsies with PMIs less than 8 hours out of our total cohort of 69, our low-PMI initiative afforded us the ability to pursue snRNA-Seq of cardiac tissue. Most prior COVID-19 autopsy studies of the heart reported PMIs of multiple days (13, 33–36); such delays compromise tissue RNA integrity of postmortem tissue for molecular analysis (37).

Consistent with 2 recent studies of cardiac microthrombi in fatal COVID-19 (13, 14), we found that the minority of COVID-19 autopsies had any focal cardiac necrosis. The infrequency of cardiac necrosis in fatal COVID-19 (9, 13, 14) contrasts with the high prevalence, magnitude, and mortality risk of cardiac troponin (cTn) elevation among critically ill COVID-19 patients (7, 52, 53), even after controlling for renal dysfunction (54, 55). This discrepancy raises the question of what, if not cardiac necrosis, leads to cTn elevation in critical COVID-19. More specifically, what is the pathophysiologic sequelae of cardiac microthrombi, if not cardiomyocyte necrosis?

Our cell composition and single-nuclei transcriptomic data demonstrate that SARS-CoV-2 indeed affects cardiomyocytes, resulting in their dysfunction and cell death. Given the uncommon detection of focal cardiac necrosis and the lack of association between microthrombi and cardiac necrosis in our entire COVID-19 cohort, the reduced proportion of cardiomyocytes in COVID-19 samples suggests that cardiomyocyte loss is due to non-necrotic cell death. Additional non-necrotic causes of serum cTn elevation, such as increased cardiomyocyte membrane permeability and vesicular release from cardiomyocytes (56, 57), may also be involved but are beyond the scope of our current study. We found no association between ventricular SARS-CoV-2 viral load and peak serum hs-cTnT, suggesting that acute cardiac injury is likely an indirect effect of COVID-19. Moreover, no evidence of SARS-CoV-2 infection of cardiomyocytes emerged from our snRNA-Seq analysis, further pointing toward an indirect mechanism of cardiomyocyte injury. Our snRNA-Seq data also put forth the possibility that cardiomyocyte injury in COVID-19 may be due to upregulation of cytokine signaling by macrophages within the heart. Upregulated macrophage cytokine signaling may also lead to cardiac pericyte cell death or pericyte-myofibroblast transition in COVID-19. Relative to reference controls, COVID-19 samples had a lower proportion of pericytes and lacked any transcriptomic evidence of SARS-CoV-2 infection. Further studies are needed to determine the mechanistic details of cardiomyocyte and pericyte cell loss in critical COVID-19.

Our foremost discovery is the identification of specific prothrombotic, antifibrinolytic, and immune-activating signaling of cardiac fibroblasts in microthrombi-positive COVID-19 hearts. We identified in COVID-19 samples profibrotic responses, which can also promote in situ microthrombi formation. The most notable of such responses are ECM deposition pathways that include secreted proteins with dual prothrombotic/antifibrinolytic and innate immune response functions. Moreover, the greater number of receptor-ligand interactions involving fibroblasts in microthrombi-positive versus microthrombi-negative COVID-19 samples highlights the significance of fibroblast signaling in the pathophysiology of COVID-19–associated cardiac microthrombi. This fibroblast prothrombotic signature may even be shared by other microthrombi-positive organs of critically ill COVID-19 patients. However, the universality of the fibroblast prothrombotic signature is unknown. Prior single-cell/snRNA-Seq studies of COVID-19 autopsy tissues either examined only COVID-19 organs or compared COVID-19 with non–COVID-19 organs. To our knowledge, our study is the first to compare the single-cell transcriptome of a COVID-19 organ according to a specific histological phenotype.

Whether the transcriptomic signatures of cardiac fibroblasts we identified are the cause or consequence of cardiac microthrombi in critical COVID-19 requires additional mechanistic studies. Regardless, both scenarios have significant implications with respect to therapeutic intervention. If causal, fibroblast activation and signaling would be an important therapeutic target for mitigating microthrombi formation in acute COVID-19. If the transcriptomic signature of cardiac fibroblasts is a result of cardiac microthrombi, countering the related pathways may prevent or treat chronic cardiac sequelae (e.g., cardiac fibrosis, microvascular disease) in survivors of severe or critical COVID-19. In either case, our overall findings show the possibility that small molecule inhibition of proteins encoded by fibroblast regulator genes (e.g., *FGFR1*, *STAT3*) may be a novel approach to the clinical management of COVID-19 cases, be it in the acute or postacute phase of critical illness. Such therapeutic potential warrants further mechanistic investigation into the role of fibroblasts in the pathophysiology of cardiac microthrombi and immunothrombosis in critical COVID-19.

### Limitations.

Patients were hospitalized prior to FDA approval of COVID-19 therapies, and our initial local practice, as of April 15, 2020, was to reserve steroids for only the most critically ill–appearing patients. Therefore, the nearly statistically significant association of steroid use with increased odds of cardiac microthrombi may reflect some confounding by an unmeasured greater severity of illness. During the study period, testing for other respiratory viruses (i.e., influenza or adenovirus) was not routine. Reassuringly, a study of suspected acute myocarditis patients earlier in the pandemic offers evidence that viral coinfection occurs less than 5% of the time (58). Our observed associations between clinical variables and presence of cardiac microthrombi may be affected by unmeasured residual confounding and missingness, though we were able to adjust for several confounders via CBPS and employed multiple imputation. The magnitude of the effect estimates may not be generalizable given our single-center study design.

While affording previously unattainable resolution of cell type–specific transcription, snRNA-Seq analyses have technological limitations, which are discussed extensively in the results and Supplemental Methods as they arise. Importantly, SARS-CoV-2 resides in the cytosol, which is removed during sample processing for snRNA-Seq. As such, we could not and did not observe SARS-CoV-2 transcripts in our analysis and must rely upon indirect evidence of viral infection of individual cell types in our analysis. Along similar lines, snRNA-Seq analysis cannot capture cellular or transcriptomic data on anuclear cells, namely platelets. We utilized an externally developed non–COVID-19 reference control; expansive conclusions regarding COVID-19 versus non–COVID-19 transcriptional signatures should be interpreted conservatively. We provide complete analysis but limit our discussion to only the most distinct findings, focusing on the features observed in internally developed microthrombi-positive versus microthrombi-negative COVID-19 comparisons. Finally, our sample size for snRNA-Seq is limited but comparable to most other snRNA-Seq analyses of pediatric (59) and adult (39, 40) human hearts.

### Conclusions.

We identified cardiac fibroblast activation as a salient feature of microthrombi-positive hearts in fatal COVID-19. Whether fibroblast activity is causative of or reactive to the formation of cardiac microthrombi remains to be determined. Still, our snRNA-Seq analysis revealed several expression signatures identifying a constellation of prothrombotic triggers at cell type resolution. Regulator genes and pathways involved in platelet function and thrombosis in fibroblasts were prominent in microthrombi-positive versus microthrombi-negative COVID-19 samples, rendering it plausible that fibroblasts are integral to the propagation of cardiac microthrombi in critical COVID-19. For future directions, we aim to elucidate the details of fibroblast activation in cardiac microthrombi pathobiology, by pursuing mechanistic studies and single-cell spatial transcriptomics with histological analysis. Further studies are also needed to assess whether therapies targeting against fibroblast activation, such as inhibitors of FGFR1 or STAT3, may potentially benefit severely ill COVID-19 patients at risk for or with cardiac microthrombi.

## Methods

### Study cohort.

We conducted a single-center prospective cohort study of hospitalized patients, aged at least 18 years, diagnosed with SARS-CoV-2 infection via RT-qPCR of nasopharyngeal swab as part of clinical care or, for persons under investigation who were suspected of having COVID-19, by postmortem RT-qPCR. This cohort represents the first 69 consecutive RT-qPCR–confirmed COVID-19 autopsies performed at CUIMC, all of whom died between March 26 and June 16, 2020.

For snRNA-Seq analysis, we selected 8 COVID-19 cases, for which snap-frozen ventricular tissue was collected at autopsy, with PMI less than 8 hours, based on the presence or absence of cardiac microthrombi and the absence of focal necrosis or myocarditis. We obtained the snRNA sequences of 6 non–COVID-19, nonfailing organ donors from the HCA (39) and processed the data as our non–COVID-19 reference cohort. The HCA decedents were organ donors who died of brain death, with primary diagnoses of stroke, suicide, or trauma. They did not have hypertension, diabetes, or any other preexisting clinical cardiac disease. All organ donors had normal left ventricular ejection fraction (>50%).

### Autopsies.

During the initial COVID-19 surge in New York City, the CUIMC Departments of Pathology and Medicine implemented a multidisciplinary initiative to lower PMI. (See Supplemental Methods for details.) At autopsy, multiple full-thickness mid–free wall samples of the left and right ventricles were collected for immediate formalin fixation and snap-freezing. For autopsies with PMI less than 8 hours, additional ventricular samples were immediately snap-frozen and stored at –80°C for subsequent snRNA-Seq analysis.

### Histology, microscopy, and quantitative analysis of histopathology.

Formalin-fixed, paraffin-embedded (FFPE) samples were cut (4 μm thick slices) and stained with H&E. Microscopy examination (Olympus BX43) of all H&E slides was performed by at least 2 anatomic pathologists. All samples were assessed for active myocarditis, defined as myocyte degeneration or necrosis in the presence of adjacent inflammatory infiltrate (60).

Immunohistology of all COVID-19 samples was also performed for this study. Four-micrometer-thick sections of left and right ventricular FFPE tissue blocks per decedent were cut and labeled using an automated staining platform (Leica Bond-III, Leica Biosystems) with the following monoclonal antibodies: C4d (Leica Biosystems, BOND Ready-to-Use Primary Antibody, clone SP91) in order to detect cell damage or, in the case of cardiomyocytes, necrosis; CD61 (Cell Marque, prediluted primary antibody, clone EP65) to identify platelet-rich microthrombi; and CD31 (Leica Biosystems, BOND Ready-to-Use Primary Antibody, clone 1A10) to verify endothelial cell identity. The target antigen was detected using either a DAB chromogen or Alkaline Phosphatase Red Chromogen. The latter was used to avoid interference from other types of brown pigment (e.g., hemosiderin or lipofuscin). All immunohistology samples were counterstained with hematoxylin. As such, cardiomyocytes could be identified by their characteristic rod-like morphology.

All slides were scanned using a Leica SCN400 slide scanner (Leica Scanner Console software v102.0.7.5). Analysis of digital slides was performed independently, in parallel, by at least 2 blinded investigators, using ImageScope (Aperio, Leica Biosystems).

Cardiomyocyte necrosis was evaluated for each ventricular sample. Focal cardiac necrosis was defined as a confluence of C4d^+^ cardiomyocytes covering an area of at least 0.05 mm^2^ but not exceeding 1 cm^2^. Scattered, individual cardiomyocyte necrosis was defined as the presence of C4d^+^ cardiomyocytes that were surrounded by C4d^–^ cardiomyocytes.

### Measurement of ventricular SARS-CoV-2 viral load.

Total RNA was extracted from FFPE tissue samples of left and right ventricles of all 69 hearts, using the Quick-RNA FFPE Miniprep Kit (Zymo Research) according to the manufacturer’s instructions. We performed RT-qPCR, using primer/probe sets for the *N1* and *N2* regions of the SARS-CoV-2 nucleocapsid gene and for the human RNase P gene (*RP*) (Integrated DNA Technologies), as described previously (61). All samples were run in triplicate. A standard curve of *N2* ranging from 10^1^ to 10^5^ viral copies was generated from the 2019-nCoV_N_Positive Control (Integrated DNA Technologies). Samples were considered positive for SARS-CoV-2 only if all 3 transcripts — *N1*, *N2*, and *RP —* were detected. (See Supplemental Methods for details.)

### Measurement of canonical gene markers of fibroblast activation in ventricular tissue.

We measured the expression levels of fibroblast activation markers *FAP* and *POSTN*, along with housekeeping genes *GAPDH* and ribosomal protein S13 (*RPS13*), in total RNA extracted from FFPE COVID-19 ventricular tissue, using the Reliance One-Step Multiplex Supermix (Bio-Rad), customized PrimePCR Probe Assays (FAP-FAM, POSTN-HEX, GAPDH-Cy5.5, RPS13-Cy5; Bio-Rad), and the CFX96 Touch Real Time PCR Detection System (Bio-Rad). All primer/probe sets were initially tested in both single and multiplex PCR reactions; Ct value differences were less than 1 cycle. Only those samples with PMI less than 24 hours were used for analysis of fibroblast activation markers. Quantitative analysis and data visualization were performed using Prism 9.0 for MacOS (GraphPad Software LLC).

### snRNA-Seq analysis.

We isolated nuclei from snap-frozen ventricular tissue, as previously described (40), and generated molecularly barcoded single-nuclei emulsions (target 8000 nuclei per device) using the 10x Genomics Chromium Controller with v3.1 Next GEM Single Cell 3′ Kit. Libraries were constructed according to manufacturer’s protocols with few modifications (Supplemental Methods) and multiplexed for sequencing on HiSeq2500 or NovaSeqS1 flow cells. All data processing was performed in the Terra Platform (https://app.terra.bio) unless otherwise noted. Postprocessing of data was accomplished using SCANPY v1.7.1 (62). We obtained FASTQ files for non–COVID-19 donor hearts from the HCA’s Heart Atlas (39) and processed them in parallel. Details regarding read alignment, filtration, quality control, marker gene and cell type identification, compositional analyses, differential expression testing, pathway enrichment, cell-cell communication, and regulator genes are in the Supplemental Methods.

### Data availability.

The processed snRNA-Seq data underlying this article are available via the Single Cell Portal (https://singlecell.broadinstitute.org/single_cell/study/SCP1464/single-nuclei-rna-sequencing-analysis-of-cardiac-microthrombi-in-fatal-covid-19, sign-in required) as well as the Gene Expression Omnibus (https://www.ncbi.nlm.nih.gov/geo/, accession GSE185457).

### Statistics.

We tested associations between the presence of ventricular SARS-CoV-2 and histopathologic findings using logistic regression while adjusting for age and sex. We examined unadjusted associations of demographic, clinical, and laboratory characteristics with the presence of cardiac microthrombi in COVID-19 hearts using 2-tailed Student’s *t*, Mann-Whitney *U*, or χ^2^ tests as appropriate. For laboratory markers with greater than 5% missingness, we used multiple imputation in unadjusted tests of association. We created separate GAMs with LOESS smoothers to test adjusted associations of the risk of cardiac microthrombi with plasma biomarkers of systemic inflammation, fibrinolysis, and cardiac injury. Due to the moderate cohort size and prevalence of microthrombi, we used CBPS to adjust for potential confounders and precision variables, which included age, sex, patient-identified race and ethnicity, BMI, duration of COVID-19 illness (defined as the time interval in days between death and documented onset of symptoms consistent with COVID-19 or initial contact with health care system for symptoms consistent with COVID-19), outpatient ACE inhibitor/ARB use, outpatient antiplatelet therapy, and inpatient administration of corticosteroids, remdesivir, IL-6 receptor antagonists, and therapeutic anticoagulation. We estimated adjusted ORs using logistic regression models if there was no evidence of nonlinearity in the GAMs. Since ESR had a nonlinear association, we estimated ORs for each quartile increase in biomarker concentration. We also examined associations of treatments with cardiac microthrombi using logistic regression with CBPS to adjust for age, sex, race and ethnicity, BMI, and duration of COVID-19 illness, remdesivir, and IL-6 receptor antagonists. Statistical significance was defined by a 2-sided *P* < 0.05. Analyses were performed using SAS (v9.4, SAS Institute) and R (v3.5.1).

### Study approval.

This study was approved by the Institutional Review Board (IRB) of CUIMC. Next of kin provided informed consent for autopsy, which was documented in the electronic medical record. (Supplemental Methods). Clinical and autopsy data were manually abstracted from the electronic medical record and deidentified, as approved by the CUIMC IRB (IRB-AAS9835). Per the Code of Federal Regulations 45 CFR 46.102, additional histological and molecular analyses of postmortem tissue were exempt from IRB evaluation (IRB-AAT0135).

## Author contributions

EJT and NRT conceptualized and designed the research project. EJT directed the overall study and protocolized and coordinated the low-PMI autopsy initiative. HH helped prepare tissue samples. NU led the IRB-approved protocol for clinical data collection. MIB, SG, and RSZ collected clinical data. GZ performed immunohistology. CCM, MJS, EJT, NF, and IR reviewed and analyzed histology. MRB, CM, and BR designed the statistical analysis of clinical data. ZZ performed statistical analysis of clinico-histological data. CT and AC performed RT-qPCR experiments. CT analyzed RT-qPCR data. RDP prepared samples for snRNA-Seq. MLH and NRT processed and analyzed snRNA-Seq data. DB, MBL, MSM, JWM, and ACU provided resources. NF, MLH, MIB, CT, CM, and MRB drafted manuscript sections. NF, MLH, CT, ZZ, MIB, NRT, and EJT prepared figures. All authors either drafted or edited manuscript sections. EJT and NRT wrote and critically revised the manuscript. All authors approved the final manuscript submission. MIB, MLH, and NF share co–first authorship as each was responsible for major project components. The order of co–first authors was determined by the temporal order of research contribution and agreed upon by the co–first authors. Co–first authors have the right to list themselves first for purposes of their curriculum vitae and biosketch.

## Supplementary Material

Supplemental data

Supplemental table 7

Supplemental table 10

Supplemental table 11

Supplemental table 12

Supplemental table 13

## Figures and Tables

**Table 1:**
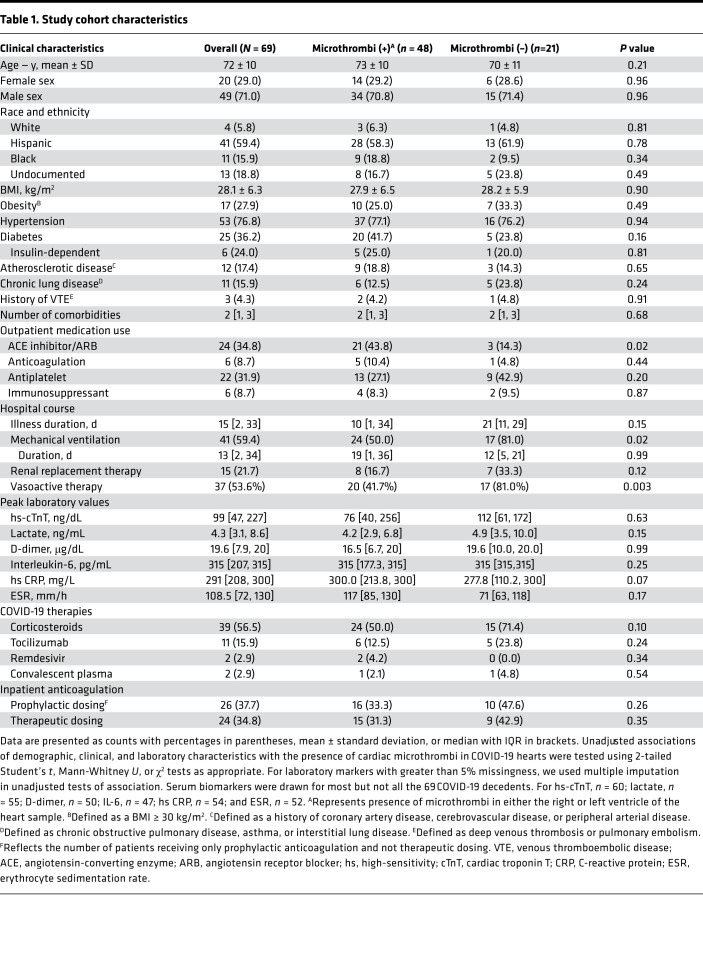
Study cohort characteristics

## Version 1 12/14/2021

In-Press Preview

## Version 2 01/25/2022

Electronic publication
