# Supplementary material for: Clinico-histopathologic and single-nuclei RNA-sequencing insights into cardiac injury and microthrombi in critical COVID-19
Source: JCI Insight. 2022 Jan 25;7(2):e154633. doi: 10.1172/jci.insight.154633 (PMC8855793; doi:10.1172/jci.insight.154633)
Supplement: Supplemental data [file jciinsight-7-154633-s107.pdf]

------------------ **Supplemental Appendix** ------------------

Clinico-histopathologic and single nuclei RNA sequencing insights into cardiac injury and microthrombi in critical COVID-19

Michael I. Brener[1]†, Michelle L. Hulke[2]†, Nobuaki Fukuma[1]†, Stephanie Golob[3], Robert Zilinyi[1], Zhipeng Zhou[4], Christos Tzimas[1], Ilaria Russo[1], Claire McGroder[5], Ryan Pfeiffer[2], Alexander Chong[6], Geping Zhang[7], Daniel Burkhoff[4], Martin B. Leon[1,4], Mathew Maurer[1], Jeffrey W. Moses[1,4,8], Anne-Catrin Uhlemann[6], Hanina Hibshoosh[7], Nir Uriel[1], Matthias J. Szabolcs[7], Björn Redfors[4], Charles C. Marboe[7], Matthew R. Baldwin[5], Nathan R. Tucker[2,9‡*], Emily J. Tsai[1‡*]

[1]Division of Cardiology, Columbia University Irving Medical Center, New York, NY; [2]Masonic Medical Research Institute, Utica, NY; [3]Department of Medicine, Columbia University College of Physicians & Surgeons, New York, NY; [4]Cardiovascular Research Foundation, New York, NY; [5]Division of Pulmonary, Allergy & Critical Care Medicine, Columbia University Irving Medical Center, New York, NY; [6]Division of Infectious Diseases and [7]Department of Pathology and Cell Biology, Columbia University Irving Medical Center, New York, NY; [8]St. Francis Hospital Heart Center, Roslyn, NY; [9]Cardiovascular Disease Initiative, Broad Institute of MIT and Harvard, Cambridge, MA.

[†]Drs. Brener, Hulke, and Fukuma all contributed equally as co-first authors.

[‡]Drs. Tsai and Tucker contributed equally as co-senior authors.

*Addresses for correspondence:

Emily J. Tsai, Division of Cardiology, Columbia University Vagelos College of Physicians & Surgeons, VP&S 8-510, 630 West 168th St, New York, NY 10032, Phone +01-212-305-3409; et2509@cumc.columbia.edu.

Nathan R. Tucker, Masonic Medical Research Institute, 2150 Bleecker St, Utica, NY 13501, Phone +01-315-624-7476, ntucker@mmri.edu

Items	Pages
**Supplemental Methods**	3
**Supplemental Tables**	
Table 1: Cardiac histopathology and ventricular viral load of COVID-19 decedents	9
Table 2: Association between microthrombi and other acute histopathologic features at the ventricular level	10
Table 3: Association between detectable SARS-CoV-2 and acute histopathologic features at the ventricular level	11
Table 4: Association between cardiac microthrombi and ESR as a categorical variable	12
Table 5: Clinical characteristics of COVID-19 (+) snRNAseq subsets	13
Table 6: Sample level quality control metrics for snRNAseq	14
Table 7: Marker genes by cell type for snRNAseq	15
Table 8: Compositional analysis from snRNAseq using scCODA comparing COVID-19(+) vs. COVID-19(-) reference samples	16
Table 9: Compositional analysis from snRNAseq using scCODA comparing COVID-19(+) samples with vs. without microthrombi	17
Table 10: Differentially expressed genes identified by snRNAseq	18
Table 11: Gene set enrichment analysis by cell type and condition using ReactomeDB	18
Table 12: Cell-cell communication as assessed through CellphoneDB	18
Table 13: Regulator genes identified by Genewalk for COVID-19 (+) vs. (-) and microthrombi (+) vs. microthrombi (-) COVID-19 subsets	18
**Supplemental Figures**	
Figure 1: Forest plot of the association between outpatient and in-hospital treatments and cardiac microthrombi	19
Figure 2: Principal components analysis of sample level transcript abundance	20
Figure 3: Identification and removal of low-quality nuclei in snRNAseq data	21
Figure 4: Volcano plots displaying differential expression results for presence of COVID-19 and for presence of microthrombi	22
Figure 5: Genewalk regulators for COVID-19(+) vs. COVID-19(-) reference samples	23
Figure 6: Ontology analysis for select regulator genes identified by Genewalk	24
Figure 7: Ventricular expression of *FAP* and *POSTN* in COVID-19(+) samples with vs. without microthrombi (PMI<24 hrs)	25
**Supplemental References**	26

Supplemental Methods

Low Post-Mortem Interval (PMI) Initiative

During the initial COVID-19 surge in New York City, the Columbia University Irving Medical Center (CUIMC) Departments of Pathology and Medicine implemented a multi-disciplinary initiative to lower post-mortem interval (PMI). Clinicians directly involved in the care of decedents obtained autopsy consent from next-of-kin at the time of death notification or shortly thereafter. Witnessed verbal consent was approved and accepted by New York Presbyterian-CUIMC since all hospital visitations were prohibited during this period. Documentation of informed autopsy consent was protocolized for the electronic medical record; a copy of the document was sent to the decedents' next-of-kin by electronic or postal mail. Timely transfer to the autopsy suite was coordinated immediately upon attainment of autopsy consent. Infographics, cloud-based resources, and multiple daily HIPAA-compliant text communications to frontline clinicians were created and used to support the low PMI initiative.

Measurement of ventricular SARS-CoV-2 viral load

Total RNA was extracted from formalin-fixed paraffin-embedded (FFPE) tissue samples of left (LV) and right ventricles (RV) of all 69 hearts, using the Quick-RNA FFPE Miniprep Kit (Zymo Research, Irvine, CA) according to the manufacturer's instructions. RNA elution was performed with nuclease free double-distilled H_2O at a final volume of 75 µl. We performed quantitative reverse transcription-polymerase chain reaction (RT-qPCR), using primer/probe sets for the *N1* and *N2* regions of the SARS-CoV-2 nucleocapsid gene and for the human RNase P gene (*RP*) (Integrated DNA Technologies), as described previously (1). All samples were run in triplicate. A standard curve of *N2* ranging from 10^1-10^5 viral copies was generated from the 2019-nCoV_N_Positive Control (Integrated DNA Technologies, Coralville, IA). Samples

were considered positive for SARS-CoV-2 only if all three transcripts--- *N1*, *N2*, and *RP*--- were detected.

For each RT-qPCR reaction, we used 5 µL of the total 75 µL of extracted RNA per ventricular tissue sample. Hence, the calculated viral load of each *N2* RT-qPCR reaction was multiplied by 15 to yield the ventricular viral load of the sample. To account for potential differences in ventricular tissue sample sizes, ventricular viral loads were normalized to *RP* expression using the Δ cycle threshold (Ct) method. Specifically, a normalization ratio for *RP* expression was calculated as $2^{-\Delta Ct}$, whereby ΔCt was the difference between each sample's *RP* Ct and the mean *RP* Ct of all samples. Thus, the normalized viral load for each ventricular tissue sample was calculated by dividing the raw ventricular viral load by the normalization ratio.

RT-qPCR of canonical genes of fibroblast activation

Total RNA was extracted from formalin-fixed paraffin-embedded (FFPE) tissue samples of the RV of all COVID-19 hearts for which PMI was less than 24 hours. Total RNA was extracted using the Quick-RNA FFPE Miniprep Kit (Zymo Research, Irvine, CA) according to the manufacturer's instructions. Only those samples with detectable housekeeping genes GAPDH and RPS13 were included in summary analysis.

Data Processing of single nuclei RNA sequences

Raw base call sequencing files were de-multiplexed; FASTQ files were generated using the 10x Genomics CellRanger 4.0.0 in the Cumulus workflow (2). To remove homopolymers (A30, T30, G30, and C30) and the template switch oligo sequence (CCCATGTACTCTGCGTTGATACCACTGCTT and its complement AAGCAGTGGTATCAACGCAGA GTACATGGG), reads were trimmed using Cutadapt v2.8 (3) with default parameters. Trimmed reads were aligned to the GRCh38 pre-mRNA human reference with SARS-CoV2 annotations (NC_045512). Count matrices were generated using CellRanger Count v12.

We inspected each sample for mapping quality based on the number of mapped reads per nucleus, percentage of mapped mitochondrial reads (%mitochondrial reads), and the shape of the unique molecular identifier (UMI) decay curve. One sample (SID 69-RV) demonstrated high levels of mitochondrial reads and was removed from further analyses. The remaining samples were filtered using CellBender v2.0 (4) with default settings to remove the ambient RNA byproducts of nuclear isolation. From this, 108,016 nuclei remained.

Quality control was performed on the individual sample level. For each droplet, we calculated the ratio of reads mapping to exonic regions to total mapped reads using Scrinvex v13 (https://github.com/getzlab/scrinvex). Droplets with an exon ratio greater than the 75th percentile + IQR range were removed from downstream analyses due to increased cytoplasmic transcripts (3,182 nuclei). We also excluded droplets which contained more than one nucleus as identified by Scrublet (5) (10,866 nuclei). Samples were also filtered to remove nuclei with reads mapped to less than 200 genes (11,280 nuclei) and nuclei with greater than 5% mitochondrial reads (9,825 nuclei).

The gene list was filtered for highly variable genes (minimum mean 0.0125, maximum mean 3, minimum dispersion 0.5), using a subset of 6,255 genes for graph-based clustering. To account for variable complexity per nucleus, counts were normalized to 10,000 unique molecules per nucleus and logarithmized. Total read count and % mitochondrial reads were regressed out (Scanpy preprocessing *regress_out*); data was scaled to a maximum value of 10. Ischemic time for non-COVID-19 reference controls (6) was unavailable. Hence, we could not account for effects of ischemic time; results relating to hypoxic signaling should be interpreted with caution.

We calculated principal components from the highly variable gene subset (scanpy.tl.pca(adata, svd_solver='arpack')) and then corrected the normalized data for batch

effects using Harmonypy version 0.0.5 (7) with each sample considered as a unique batch. We used the batch-corrected PCs to calculate neighbors (scanpy.pp.neighbors(adata, n_neighbors=10, n_pcs=40)) and generate a UMAP (scanpy.tl.umap(adata)). Nuclei were then clustered using leiden clustering (scanpy.tl.leiden(adata)) at a resolution of 0.225.

Marker gene and cell type identification

Genes were ranked using a Wilcoxon rank sum test (scanpy *rank_genes_groups*) for each cell-type cluster versus all others; and $\log_2$-fold change (FC) and percentage of nuclei expressing each gene were calculated. Area under the receiver operator curve (AUC) scores were calculated for all genes within each cluster using SciKit Learn *roc_auc_score*. Genes were considered markers of a given cluster with an AUC score >0.7 or a $\log_2$-FC >0.6.

Compositional analyses

The proportion of each cell type was compared across samples using scCODA version 0.1.1 (8). Briefly, we constructed a Markov-Chain Monte Carlo model with Hamiltonian Monte Carlo sampling using cell type proportions between conditions (non-COVID-19 reference control versus COVID-19, or COVID-19 microthrombi-positive versus COVID-19 microthrombi-negative). Credible interval differences in cell type proportions were determined using spike-and-slab inclusion probabilities. Importantly, the proportion of a given cell type in a sample is governed by our ability to liberate the nuclei equivalently from the tissue and our ability to successfully identify cells compared to empty droplets. The former may be affected by cell death or increased fibrosis which our sectioning protocol is designed to mitigate. The latter is a more challenging problem for single nucleus RNA sequencing, which is influenced by the relative transcriptional complexity of various cell types, making transcript rich cell types such as cardiomyocytes and fibroblasts easy to identify with transcript poor cells such as immune cells more apt to be assigned as an empty droplet. Use of a probabilistic cell calling mechanism in CellBender(4) is used to overcome this challenge.

Differential expression testing

Differentially expressed genes (DEGs) were calculated for each major cell cluster separately using the MAST(9) pipeline. We constructed a Hurdle model (zlm(~condition + ngenes + (1 | sample_ID), sca,method='glmer', ebayes = FALSE, strictConvergence = FALSE)) with the normalized reads based on the cellular detection rate, the set condition (either donor control versus COVID-19, or COVID-19 microthrombi-positive versus COVID-19 microthrombi-negative), and biological individual. Only genes with non-zero expression in at least 15% of all nuclei were included. DEGs were identified based on an Benjamini-Hochberg false discovery rate (FDR) adjusted P-value <0.05. Importantly, each droplet is contaminated by ambient RNA, which is imperfectly removed informatically by design. Therefore, DE results should be interpreted with caution, particularly when examining non-major cell types (e.g., intracardiac neurons, lymphatic endothelial, mast cells) and genes highly expressed in those as numerous as cardiomyocytes. Genes which serve as markers of another cluster (AUC $\geq$ 0.7) were blacklisted to exclude potential differential contamination by ambient RNA as a driver of such effect. Gene lists were also compared to the secreted protein list obtained from the Human Protein Atlas (proteinatlas.org). Full lists of DE genes, including those which were blacklisted, are contained in Supplemental Table ST3.

Reactome pathway enrichment

For each major cell type, we performed gene pathway enrichment using Reactome(10) (Pathway browser version 3.7, database release 75) on DEGs with $\log_2$-FC >0.25, separated into up or downregulated genes. We also calculated pathway enrichment using the Reactome pathway from GSEA Msigdb(11) (MSigDB database v7.2). Pathways were considered enriched within a cell type if identified as such by both Reactome and GSEA Msigdb (BH FDR adjusted

P-value of ≤ 0.05). Reactome terms for all comparisons are available in Supplemental Table ST11.

Cell-Cell Communication

Cell-cell communication was tested with CellphoneDB version 2.1.7 (12) on each sample separately using normalized count data for the 9 largest cell types. Afterward, significant interactions were aggregated between microthrombi-positive and microthrombi-negative samples. Briefly, CellphoneDB identifies and compares ligand-receptor interaction pairs between cell types and compares the observed interactions to the expected interactions of a null distribution generated from randomly permuted cell labels. Default parameters were used for the analysis (10% threshold for cells expressing ligands and receptors, p-value = 0.05, 1000 iterations for generation of null distribution, curated interactions list compiled by CellphoneDB from UniProt, Ensembl, PDB, IMEx consortium, and IUPHAR).

Regulator Genes

To identify regulator genes that function within and likely drive gene networks within a biological context, we analyzed DEGs with a $\log_2$-FC ≥0.5 for each cell type using GeneWalk(13) (direction of differential expression was not incorporated). GeneWalk builds biologically relevant networks from provided gene lists, connecting genes and GO terms, and compares the network to random networks. GeneWalk was used with default parameters and an FDR-corrected P-value of 0.1. The identified regulator genes were compared to a list of druggable genes.(14) The full list of regulator genes identified is available in Supplemental Table 13.

Supplemental Table 1. Cardiac histopathology and ventricular viral load of COVID-19 decedents

COVID-19	Overall (n=69)	Microthrombi-Positive* (n=48)	Microthrombi-Negative (n=21)
Ventricular Viral load			
Detectable viral load	43 (62.3)	31 (64.5)	12 (57.1)
SARS-CoV-2 viral load* (copies)	1055 [307, 13285]	1594 [562,13285]	387 [196, 21811]
Cardiac Autopsy Findings, n(%)			
Left ventricular hypertrophy	45 (65.2)	34 (70.8)	11 (52.4)
Left ventricular dilation	18 (26.1)	16 (33.3)§	2 (9.5)
Right ventricular hypertrophy	21 (30.4)	16 (33.3)	5 (23.8)
Right ventricular dilation	37 (53.6)	27 (56.3)	10 (47.6)
Coronary atherosclerosis	39 (56.5)	26 (54.2)	13 (61.9)
Myocardial infarction	14 (20.3)	10 (20.8)	4 (19.0)
Thrombus†	2 (2.9)	2 (4.2)	0 (0.0)
Interstitial edema	5 (7.2)	3 (6.3)	2 (9.5)
Perivascular fibrosis	23 (33.3)	16 (33.3)	7 (33.3)
Interstitial fibrosis	27 (39.1)	21 (43.8)	6 (28.6)
Wavy myocytes	7 (10.1)	3 (6.3)	4 (19.0)
Contraction bands	5 (7.2)	3 (6.3)	2 (9.5)
Pericardial findings	12 (17.4)	8 (16.7)	4 (19.0)
Cardiac Histopathologic Findings‡, n(%)			
Microvascular endothelial cell damage	25 (36.2)	19 (39.6)§	6 (28.6)
Scattered individual cardiomyocyte necrosis	25 (36.2)	16 (33.3)	9 (42.8)
Focal cardiac necrosis	14 (20.3)	11 (22.9)	3 (14.3)
Focal inflammatory infiltrate	12 (17.4)	6 (12.5)	6 (28.6)
Focal myocarditis	4 (5.8)	2 (4.2)	2 (9.5)
Pulmonary Autopsy Findings, n(%)			
Diffuse alveolar damage	38 (55.1)	27 (56.3)	11 (52.4)
Pulmonary artery thrombosis	10 (14.5)	7 (14.6)	3 (14.3)
Pulmonary microvascular thrombi	42 (60.9)	31 (64.6)	11 (52.4)

Data are presented as counts with percentages in parenthesis and median with interquartile range in brackets.

* Based upon the higher value detected in either the left or right ventricle of decedent

† Intraventricular, intra-atrial, or epicardial coronary arterial thrombus

‡ Based upon detection in either left or right ventricle of decedent

§ $P<0.05$

Supplemental Table 2. Association between microthrombi and other acute histopathologic features at the ventricular level.

Dependent Variable	Independent Variable	Univariate Model		Multivariate Model	
		OR (95% CI)	p	OR (95% CI)	p
Microthrombi	Microvascular endothelial cell damage	3.44 (1.42, 8.32)	0.006	3.58 (1.46, 8.80)	0.005
Scattered individual necrotic cardiomyocytes	Microthrombi	1.00 (0.46, 2.20)	1.00	1.05 (0.47, 2.34)	0.90
Focal cardiac necrosis	Microthrombi	1.23(0.37, 4.14)	0.74	1.03 (0.30, 3.56)	0.96
Focal inflammatory infiltrate	Microthrombi	0.43 (0.14, 1.35)	0.15	0.39 (0.12, 1.25)	0.11
Focal myocarditis	Microthrombi	1.27 (0.21, 7.85)	0.80	1.30 (0.20, 8.52)	0.78

All histopathologic features listed above were identified on immunohistologic microscopy with the exception of focal inflammatory infiltrate and myocarditis, which were identified by H&E staining. Details in Supplemental Methods. Multivariate model is adjusted with age and sex.

Supplemental Table 3. Association between detectable SARS-CoV-2 and acute histopathologic features at the ventricular level

Histopathologic Feature	Univariate Model		Multivariate Model	
	OR (95% CI)	p	OR (95% CI)	p
**Microthrombi**	1.52 (0.78, 2.99)	0.22	1.62 (0.81, 3.22)	0.17
**Microvascular endothelial cell damage**	2.24 (1.00, 5.03)	0.05	2.36 (1.04, 5.35)	0.04
**Scattered individual necrotic cardiomyocytes**	1.42 (0.66, 3.04)	0.37	1.43 (0.67, 3.04)	0.35
**Focal cardiac necrosis**	0.40 (0.12, 1.27)	0.12	0.42 (0.13, 1.36)	0.15
**Focal inflammatory infiltrate or myocarditis**	0.30 (0.08, 1.03)	0.06	0.28 (0.08, 1.04)	0.06

All histopathologic features listed above were identified on immunohistologic microscopy with the exception of focal inflammatory infiltrate or myocarditis, which was identified by H&E staining. Details in Supplemental Methods. Multivariate model is adjusted with age and sex.

Supplemental Table 4. Association between cardiac microthrombi and ESR as a categorical variable.

ESR		Unadjusted OR (95% CI)	P-value	Adjusted OR (95% CI)	P-Value
Quartile	mm/hr				
1st	27.0-80.0	*Reference*			
2nd	81.0-107.1	1.70 (0.65-4.50)	0.280	0.87 (0.24-3.23)	0.840
3rd	107.2-126.0	2.78 (1.08-7.17)	0.034	3.76 (1.17-12.04)	0.026
4th	130.0-169.1	1.95 (0.77-4.91)	0.160	6.65 (1.53-28.79)	0.011

Logistic regression model was adjusted for possible confounders by calculating a covariate balancing propensity score (CBPS) and using it as a single covariable. The covariates used to calculate CBPS were: age, sex, race/ethnicity, body mass index, duration of Covid-19 illness, outpatient ACEi/ARB use, outpatient antiplatelet therapy, and inpatient administration of corticosteroids, remdesivir, interleukin-6 (IL-6) receptor antagonists, and therapeutic anticoagulation.

ESR = Erythrocyte sedimentation rate, OR = odds ratio, CI = confidence interval

Supplemental Table. Clinical characteristics of COVID-19 (+) snRNAseq subset

	Microthrombi (+) *			Microthrombi (-)			
Study ID	*05*	*39*	*61*	*19*	*45*	*51*	*66*
Baseline Characteristics							
Age – yr	83	71	58	68	65	63	69
Sex	Male	Male	Male	Male	Male	Male	Female
Race/ethnicity	Hispanic	Hispanic	Hispanic	Hispanic	Hispanic	Hispanic	n/a
Body mass index – kg/m²	24.0	34.7	28.5	32.0	29.0	34.5	23.0
Obesity†	·	✓	·	✓	·	✓	·
Hypertension	✓	✓	·	✓	✓	✓	✓
Diabetes	·	✓	✓	·	·	·	·
Insulin-dependent	·	·	✓	·	·	·	·
Atherosclerotic disease‡	·	·	·	·	·	✓	·
Chronic lung disease§	·	·	·	·	✓	·	✓
History of VTE¶	·	·	·	·	·	·	✓
Number of Comorbidities	1	2	1	1	2	2	3
Outpatient Medication Use							
ACE inhibitor/ARB	·	✓	·	·	·	·	·
Anticoagulation	·	·	·	·	·	·	·
Antiplatelet	✓	·	·	·	✓	·	·
Immunosuppressant	·	·	·	·	✓	·	·
Clinical Course							
Duration of illness – days	9	26	57	26	24	21	40
Mechanical ventilation	·	✓	✓	✓	✓	✓	·
Duration – days	·	0	57	21	7	9	·
Renal replacement therapy	·	·	✓	·	✓	·	·
Vasoactive support	·	·	✓	✓	✓	✓	·
Laboratory Studies, peak values							
hs Troponin T, ng/dL	410	24	292	542	212	30	61
Lactate, ng/mL	1.7	4.2	4.5	3.1	10.1	11.1	3.9
D-dimer, μg/dL	1.01	5.30	20.00	20.00	20.00	20.00	10.00
Interleukin-6, pg/mL	207.0	315.0	315.0	315.0	315.0	273.0	108.0
hs C-reactive protein, mg/L	124	300	300	278	234	109	278
ESR, mm/hr	35	109	130	63	39	27	130
Covid-19 Therapies							
Corticosteroids	·	✓	✓	✓	✓	✓	✓
Tocilizumab	✓	✓	·	✓	✓	·	·
Remdesivir	·	·	·	·	·	·	·
Convalescent plasma	·	·	·	·	✓	·	·
In-hospital Anticoagulation							
Prophylactic dosing	✓	✓	✓	·	✓	✓	·
Therapeutic dosing	·	·	·	✓	·	·	✓

n/a=not available (undocumented)
✓ Represents presence of trait/therapy/finding
· Represents absence of trait/therapy/finding
VTE = venous thromboembolic disease
ACE = angiotensin converting enzyme
ARB = angiotensin receptor blocker
hs = high-sensitivity
ESR = erythrocyte sedimentation rate
* Detected on IHC of right ventricle

† Body mass index (BMI) ≥30 kg/m²
‡ History of coronary artery disease, cerebrovascular disease, or peripheral arterial disease
§ Chronic obstructive pulmonary disease, asthma, or interstitial lung disease
¶ Deep venous thrombosis or pulmonary embolism
ǁ Reflects the number of patients receiving only prophylactic and not therapeutic anticoagulation

Supplemental Table 6. Sample level quality control metrics for snRNAseq

Study ID	Estimated Number of Nuclei	Mean Reads per Nucleus	Median Genes per Nucleus	Number of Reads	Total Genes Detected	Median UMI Counts per Nucleus	Microthrombi	Included in Analysis	Post QC Number of Nuclei
**05**	9,575	10,223	791	97,892,749	28,791	1,122	Positive	TRUE	7208
**39**	3,497	56,326	758	196,972,045	26,402	1,285	Positive	TRUE	1846
**51**	9,886	21,626	1,910	213,804,137	35,115	3,979	Negative	TRUE	5787
**19**	14,427	34,978	2,072	504,630,120	38,265	3,735	Negative	TRUE	9947
**45**	9,683	31,419	1,670	304,235,656	35,936	3,092	Negative	TRUE	6851
**66**	9,150	11,807	1,607	108,041,509	33,527	3,059	Negative	TRUE	7840
**61**	5,412	15,253	1,158	82,552,819	31,457	1,871	Positive	TRUE	4014
**69**	3,905	13,434	266	52,460,970	26,291	416	Positive	FALSE	0

Study ID: COVID-19(+) sample ID

Estimated Number of Nuclei: Number of droplets called as nuclei from CellRanger pipeline

Mean Reads per Nucleus: Average number of mapped reads per nucleus

Median Genes per Nucleus: Median number of genes detected in nuclei

Number of Reads: Total number of reads with multiplexing index matching a given sample

Total Genes Detected: Total unique gene IDs detected across all nuclei

Median UMI Counts per Nucleus: Median number of unique transcript molecules detected per nucleus

Microthrombi: Detection of cardiac microthrombi by immunohistochemistry (CD61 staining of corresponding ventricular tissue)

Included in Analysis: Use of this sample in downstream analysis pipeline

Post-QC Number of Nuclei: Number of nuclei retained following filtering for aberrant mitochondrial reads, intron/exon ratio, and doublet score
